# Targeting Macular Pigment in Intermediate Age-Related Macular Degeneration: Oral Supplementation Versus Transscleral Iontophoresis in a Prospective Pilot Study

**DOI:** 10.3390/jcm15093188

**Published:** 2026-04-22

**Authors:** Michele Rinaldi, Gilda Cennamo, Maria Laura Passaro, Flavia Chiosi, Fulvia De Falco, Alfonso D’Alessandro, Diego Strianese, Ciro Costagliola

**Affiliations:** 1 Department of Neurosciences, Reproductive Sciences and Dentistry, University of Naples “Federico II”, 80131 Naples, Italy; michrinaldi@libero.it (M.R.); fulvia.defalco@yahoo.com (F.D.F.); alfodal@yahoo.com (A.D.); strianes@unina.it (D.S.); ciro.costagliola@unina.it (C.C.); 2 Department of Medicine and Health Sciences “V. Tiberio”, University of Molise, 86100 Campobasso, Italy; 3 Department of Ophthalmology, Azienda Ospedaliera dei Colli-Ospedale Monaldi, 80131 Naples, Italy; flaviachiosi@yahoo.it

**Keywords:** age-related macular degeneration, macular pigment optical density, iontophoresis, carotenoids, lutein, zeaxanthin, intermediate AMD, OCT

## Abstract

**Background/Objectives**: Macular pigment optical density (MPOD) represents a biomarker of retinal antioxidant status in intermediate age-related macular degeneration (iAMD). Strategies aimed at increasing macular carotenoid availability may contribute to disease stabilization. This study evaluated the effects of oral supplementation and transscleral iontophoresis on MPOD and retinal parameters in iAMD. **Methods**: This prospective, non-randomized pilot study included 60 eyes of 60 patients with intermediate AMD enrolled at the Eye Clinic of the University of Naples Federico II between July 2024 and May 2025 (ClinicalTrials.gov NCT06465342). Patients received either oral carotenoid supplementation (*n* = 30) or transscleral iontophoresis (n = 30). Best-corrected visual acuity (BCVA), central macular thickness (CMT), and MPOD measured by one-wavelength reflectometry ( Visucam 200; Carl Zeiss Meditec, Jena, Germany) were assessed at baseline and 6 months. **Results**: BCVA remained stable in both groups without significant changes (*p* > 0.05). MPOD significantly increased in the iontophoresis group (0.40 ± 0.11 to 0.49 ± 0.12, *p* < 0.001) with no statistically significant difference between them (*p* = 0.09). CMT showed a mild, non-significant increase in both groups (*p* > 0.05). No adverse events were observed. **Conclusions**: Both oral supplementation and transscleral iontophoresis were associated with a significant increase in MPOD while preserving visual acuity in intermediate AMD. Within the limitations of this non-randomized pilot study, transscleral iontophoresis produced MPOD changes comparable to those observed with oral supplementation. These findings are exploratory and support further investigation of localized delivery strategies in larger, randomized trials.

## 1. Introduction

Age-related macular degeneration (AMD) is a chronic, progressive retinal disease and remains one of the leading causes of central vision loss in the elderly population worldwide [1,2,3]. In the intermediate stage, characterized by large drusen and/or retinal pigment epithelium abnormalities in the absence of macular neovascularization or central geographic atrophy, there is still a window for intervention. At this stage, strategies aimed at slowing disease progression are of particular interest, as structural damage is not yet advanced and may still be modifiable [4].

Macular pigment optical density (MPOD) reflects the concentration of the xanthophyll carotenoids lutein, zeaxanthin, and meso-zeaxanthin in the central retina and is commonly used as a biomarker of macular antioxidant status [5,6]. These carotenoids preferentially accumulate in the foveal region, where they act as short-wavelength light filters and help counteract oxidative stress [7]. Several studies have shown that MPOD levels tend to be reduced in patients with AMD, suggesting an association between lower macular pigment and the presence or progression of the disease. While these observations are consistent with the hypothesis that reduced macular pigments may contribute to increased vulnerability of the macula, a causal relationship has not been definitively established [8,9].

Oral supplementation with lutein and zeaxanthin has been shown to increase MPOD in both healthy individuals and patients with early or intermediate AMD. In addition, higher dietary intake of these carotenoids has been associated with a lower risk of progression to advanced disease [10]. However, the retinal bioavailability of orally administered carotenoids is variable. Factors such as intestinal absorption, systemic metabolism, lipoprotein transport, and individual nutritional status can influence the amount of lutein that ultimately reaches the retina. As a result, oral supplementation may not always achieve consistent or optimal retinal concentrations [11].

For this reason, alternative strategies aimed at improving retinal delivery of carotenoids have been explored. Transscleral iontophoresis is a non-invasive technique that uses a low-intensity electrical current to enhance the penetration of molecules across the sclera, potentially allowing more direct delivery to the posterior segment of the eye [12]. Experimental and early clinical data suggest that this approach may increase intraocular lutein availability and represent a viable route for targeted retinal delivery [13].

In this context, the present prospective, non-randomized pilot study was designed to evaluate and compare the effects of oral macular carotenoid supplementation and transscleral lutein iontophoresis on MPOD in patients with intermediate AMD.

## 2. Materials and Methods

### 2.1. Study Design and Setting

This prospective, non-randomized pilot study was conducted at the Eye Clinic of the University of Naples “Federico II” between July 2024 and May 2025. The study adhered to the tenets of the Declaration of Helsinki and was approved by the Territorial Ethics Committee of the Campania Region, Italy (Decree No. 65, 16 June 2023). The study was registered at ClinicalTrials.gov (Identifier: NCT06465342). Written informed consent was obtained from all participants prior to enrollment, with strict adherence to confidentiality and privacy regulations.

### 2.2. Study Population

Patients affected by intermediate age-related macular degeneration (iAMD), classified according to the Beckman classification [4], were prospectively enrolled. Inclusion criteria were: age ≥ 55 years; presence of large drusen (≥125 μm) and/or retinal pigment epithelium abnormalities; best-corrected visual acuity (BCVA) ≥ 0.52 LogMAR; absence of macular neovascularization or foveal geographic atrophy. In addition to the Beckman classification criteria, the baseline structural characteristics of intermediate AMD were assessed using multimodal imaging. Particular attention was given to drusen characteristics, including size and confluence, as well as to alterations of the retinal pigment epithelium (RPE), such as hyper- or hypopigmentation. Optical coherence tomography (OCT) was used to evaluate structural biomarkers associated with disease progression, including the presence of hyperreflective foci, drusen morphology, and the integrity of the outer retinal layers. Exclusion criteria included: previous intravitreal therapy; other retinal or optic nerve diseases; significant media opacities; ocular surgery within the previous 6 months; and systemic diseases potentially affecting retinal structure or function. Participants were also excluded if they had signs of any other active retinal disease, such as retinal vascular (e.g., diabetic retinopathy or retinal vein occlusion) or vitreoretinal (e.g., vitreomacular traction syndrome or epiretinal membrane) diseases; corneal lesions, scars, or abrasions; significant opacities in the study eye; or a medical history of ocular hypertension, glaucoma, macular pucker, optic neuropathy, or severe dry eye syndrome affecting the study eye. Further exclusion criteria were active ocular infections or inflammation, use of medications known to impact ocular health (such as digoxin, quinolines, chlorpromazine, tamoxifen, or thiazide diuretics), and intake of lutein, zeaxanthin, or similar ocular supplements within four weeks prior to screening. Additional exclusion criteria included hypersensitivity to components of the medical device, a diagnosis of type I diabetes or a history of cerebrovascular accidents, uncontrolled hypertension or cardiovascular disease deemed incompatible with study participation, heavy smoking (more than 20 cigarettes per day), significant alcohol consumption (more than two standard drinks per day), or concurrent use of menopausal hormone replacement therapy. Pregnant or breastfeeding women, those intending to conceive during the study, or individuals unable to adhere to effective contraceptive measures were also excluded. Only one eye per patient was included in the analysis. A total of 60 eyes from 60 consecutive patients were enrolled and assigned to one of two treatment cohorts: oral supplementation (*n* = 30) or transscleral iontophoresis (n = 30). Allocation to treatment groups was not randomized. Patients were assigned to either oral supplementation or transscleral iontophoresis based on clinical judgment, treatment availability, and patient preference. No matching or stratification procedures were applied. Given the non-randomized design, potential selection bias cannot be excluded. No formal sample size calculation was performed, as this was designed as an exploratory pilot study. The sample size was determined pragmatically based on the number of eligible patients during the study period. Therefore, this study may be underpowered to detect small between-group differences.

### 2.3. Treatment Protocols

Patients assigned to the oral supplementation group received daily oral supplementation containing macular carotenoids, including lutein (10 mg/die) and zeaxanthin (2 mg/die), in combination with antioxidant compounds, in accordance with standard clinical practice for the management of intermediate age-related macular degeneration. Patients were instructed to maintain regular daily intake throughout the study period.

Patients assigned to the iontophoresis group underwent a single session of transscleral iontophoresis aimed at enhancing intraocular delivery of macular carotenoids. All procedures were performed under topical anesthesia using a standardized treatment protocol with predefined current intensity and treatment duration, in accordance with device specifications and previously published clinical protocols [13]. For scleral iontophoresis, one drop of topical anesthetic (0.4% oxybuprocaine, Novesina, Novartis, Basel, Switzerland) was instilled in the study eye. After cleansing the forehead with 70% alcohol, the return electrode was positioned on the skin. The power generator (K-IONO, Offthealth SpA, Florence, Italy) was then connected to a dedicated ocular applicator, which was placed on the eye and filled with a liquid lutein formulation (Lipo+, Offthealth SpA, Florence, Italy). A constant current of 2.5 mA was delivered for 4 min. At the end of the application, the device was removed, the ocular surface was irrigated with balanced saline solution, and patients remained supine for an additional 5 min before leaving the clinic.

### 2.4. Ophthalmological Examination

Patients were evaluated at baseline, 3 months, and 6 months. The 3-month visit was primarily intended for safety monitoring and assessment of treatment adherence, and was not included in the formal statistical analysis, which focused on baseline and 6-month outcomes. Best-corrected visual acuity was assessed using Early Treatment Diabetic Retinopathy Study (ETDRS) charts and converted to logarithm of the minimum angle of resolution (logMAR) units. Slit-lamp biomicroscopy, intraocular pressure measurement, and dilated fundus examination were performed at each visit. Macular imaging was obtained with enhanced depth imaging optical coherence tomography (EDI-OCT; Spectralis, Heidelberg Engineering Inc., Heidelberd, Germany), after pharmacologic mydriasis in all participants to improve signal quality and ensure reliable retinal layer segmentation. Central macular thickness was derived automatically from the central subfield using the manufacturer’s analysis software.

MPOD was evaluated with a fundus camera incorporating a single-wavelength reflectometry module (Visucam 200; Carl Zeiss Meditec, Jena, Germany). Examinations were performed by a single experienced operator according to a standardized acquisition protocol in order to limit measurement variability. Subjects were positioned with the head stabilized on the chin and forehead rests, and internal fixation was used to maintain image centration. Following instillation of 1% tropicamide, color fundus images covering 45° were acquired approximately 30 min later.

MPOD analysis was conducted over a 30° region within the recorded fundus image. The device’s automatic exposure settings were used (flash intensity 12 and autofocus enabled). Both mean MPOD, reflecting the average xanthophyll density across the selected area, and MPOD volume were calculated using the dedicated software and are reported in density units (du).

### 2.5. Outcome Measures

The primary outcome was the change in MPOD from baseline to 6 months.

Secondary outcome measures included changes in best-corrected visual acuity, variations in central macular thickness, and treatment safety and tolerability over the study period.

### 2.6. Statistical Analysis

Statistical analyses were performed using SPSS software and IBM SPSS Statistics for Windows, Version 26.0 (IBM Corp., Armonk, NY, USA). Statistical analyses were restricted to baseline and 6-month data, in line with the exploratory design of the study. Normality of data distribution was assessed using the Shapiro–Wilk test. Within-group comparisons between baseline and follow-up values were performed using paired *t*-tests. Between-group comparisons of absolute values or changes from baseline were analyzed using independent-sample *t*-tests. All statistical tests were two-sided, and a *p*-value < 0.05 was considered statistically significant. In addition to *p*-values, effect sizes (Cohen’s d) and 95% confidence intervals were calculated for the main outcomes to provide a more comprehensive interpretation of the results. Effect sizes were interpreted according to standard conventions (small: 0.2; medium: 0.5; large: 0.8). Given the exploratory design and the relatively small sample size, a simplified statistical approach based on paired and independent *t*-tests was adopted.

## 3. Results

### 3.1. Baseline Characteristics

Sixty patients (60 eyes, 100%) completed the 6-month follow-up. Thirty eyes were included in the oral supplementation group, and thirty were included in the transscleral iontophoresis group. Baseline demographic and clinical characteristics were comparable between the two cohorts (Table 1). Mean age was 71.4 ± 6.8 years in the supplementation group and 72.1 ± 7.2 years in the iontophoresis group. Baseline BCVA, MPOD, and central macular thickness did not differ significantly between groups (all *p* > 0.05). Both groups showed a significant increase in MPOD over time, with large within-group effect sizes (Cohen’s d = 1.20 for the supplementation group and d = 1.50 for the iontophoresis group).

The between-group comparison of MPOD change showed a moderate effect size (Cohen’s d = 0.55), although statistical significance was not reached (*p* = 0.09).

### 3.2. Functional Outcomes

BCVA remained stable throughout follow-up in both groups, with no statistically significant change at 6 months. In the oral supplementation group, the mean BCVA was 0.18 ± 0.09 logMAR at baseline and 0.17 ± 0.10 logMAR at 6 months (*p* = 0.42). In the iontophoresis group, the mean BCVA was 0.17 ± 0.08 logMAR at baseline and 0.16 ± 0.09 logMAR at 6 months (*p* = 0.38). No statistically significant differences in BCVA were observed between groups at 6 months (*p* = 0.67).

**Figure 1:**
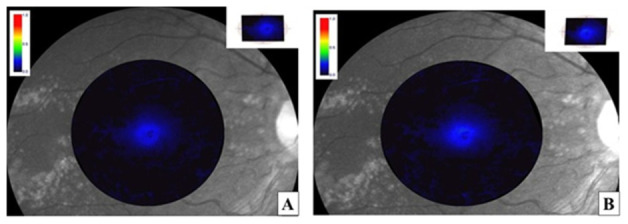
Right eye of a 72-year-old male patient affected by intermediate age-related macular degeneration (iAMD). Baseline macular pigment optical density (MPOD) values measured using Visucam 200 (Carl Zeiss Meditec) (**A**) appeared significantly increased after 6 months of treatment with oral lutein supplementation (**B**).

**Figure 2:**
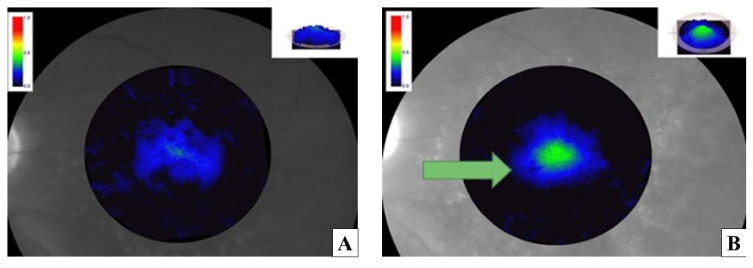
Left eye of a 69-year-old male patient affected by intermediate age-related macular degeneration (iAMD) at baseline (**A**) and after transscleral iontophoresis treatment (**B**). The green arrow shows a marked increase in macular pigment optical density (MPOD) after treatment.

**Figure 3:**
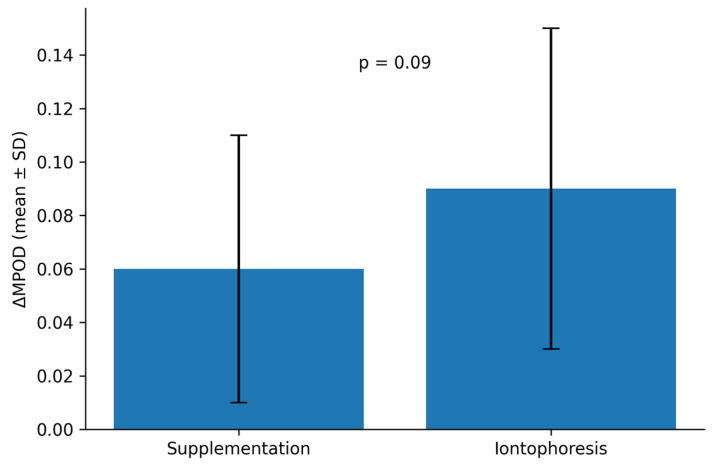
Change in macular pigment optical density (ΔMPOD) from baseline to 6 months. Mean ΔMPOD values in the oral supplementation and iontophoresis groups. A greater numerical increase was observed in the iontophoresis group; however, the between-group difference was not statistically significant (*p* = 0.09). Data are presented as mean ± standard deviation.

MPOD significantly increased from baseline to 6 months in both treatment cohorts. In the oral supplementation group, MPOD increased from 0.41 ± 0.10 at baseline to 0.47 ± 0.11 at 6 months, corresponding to a mean change of +0.06 ± 0.05 (*p* = 0.003). In the iontophoresis group, MPOD increased from 0.40 ± 0.11 at baseline to 0.49 ± 0.12 at 6 months, with a mean change of +0.09 ± 0.06 (*p* < 0.001). The between-group difference in MPOD change was not statistically significant (*p* = 0.09). Figure 1, Figure 2 and Figure 3.

CMT showed a mild increase over time in both groups, without reaching statistical significance. In the oral supplementation group, mean CMT increased from 276 ± 21 µm at baseline to 281 ± 23 µm at 6 months (*p* = 0.21). In the iontophoresis group, mean CMT increased from 279 ± 24 µm to 284 ± 25 µm over the same period (*p* = 0.18). No statistically significant differences in CMT were observed between groups at 6 months (*p* = 0.73).

The lower panels show the baseline image (C) and the image after 6 months of lutein administration by iontophoresis (D). An increased signal intensity in the central macular area is observed after treatment, particularly evident in the iontophoresis image.

### 3.3. Safety Profile

No serious ocular or systemic adverse events were reported during the study period. At each visit, patients were systematically asked about ocular discomfort, pain, photophobia, redness, blurred vision, and systemic symptoms, and a complete ophthalmic examination was performed to detect potential treatment-related complications. Minor and transient events, such as mild conjunctival hyperemia or foreign body sensation, were specifically monitored. All treatments were well tolerated, and no clinically relevant safety concerns or procedure-related complications emerged during follow-up.

## 4. Discussion

In this prospective, non-randomized pilot study involving patients with intermediate age-related macular degeneration (AMD), both oral macular carotenoid supplementation and transscleral lutein iontophoresis resulted in a significant increase in macular pigment optical density (MPOD) over a 6-month follow-up. Best-corrected visual acuity (BCVA) and central macular thickness (CMT) remained stable in both groups, and no relevant treatment-related adverse events were observed. These findings confirm that macular pigment can be effectively enhanced through different delivery strategies in this patient population and reinforce the concept that MPOD is a modifiable parameter reflecting retinal carotenoid status and the oxidative stress buffering capacity of the macular region [8,14,15,16,17,18,19,20,21,22].

MPOD is widely recognized as a modifiable clinical biomarker of macular carotenoid content and foveal antioxidant status [8,16,18]. The results observed in the oral supplementation group are consistent with previous clinical trials demonstrating that lutein and zeaxanthin supplementation leads to gradual and significant increases in MPOD and, in some cases, to improvements in functional outcomes such as contrast sensitivity and mesopic visual performance [19,20,21,23]. These effects have been reported in both early and intermediate AMD and are generally interpreted as reflecting enhanced photoreceptor resilience and reduced cumulative oxidative damage at the fovea [14,15,22,24]. Importantly, MPOD represents a surrogate biomarker of macular carotenoid status rather than a direct measure of visual function. Although a significant increase in MPOD was observed in both treatment groups, no corresponding improvement in BCVA was detected over the 6-month follow-up. This may reflect the relatively preserved baseline visual acuity in intermediate AMD and the limited sensitivity of BCVA to detect subtle functional changes. Therefore, the clinical relevance of MPOD enhancement should be interpreted with caution.

From a mechanistic perspective, the increase in MPOD observed in both treatment arms is consistent with the known biological roles of lutein and zeaxanthin in the macula. These xanthophyll carotenoids preferentially accumulate in the Henle fiber layer and inner plexiform layer, where they act as short-wavelength light filters and potent scavengers of reactive oxygen species generated by chronic light exposure and high metabolic demand [14,15,16]. Experimental and clinical evidence suggests that macular carotenoids contribute to the stabilization of photoreceptor outer segment membranes, modulation of local inflammatory signaling, and protection of the retinal pigment epithelium from photo-oxidative damage, thereby providing a plausible link between MPOD enhancement and improved functional resilience of the fovea [8,18,25].

Within this framework, transscleral iontophoresis represents a rational strategy for targeted ocular delivery. Lutein encapsulated in liposomal aggregates can traverse the sclera and ciliary body to reach the peripheral retina. Once delivered, these aggregates may exert local protective effects and subsequently diffuse toward the macular region, a process potentially facilitated by the applied electric field and associated electrophoretic transport [26]. In the present study, although no statistically significant difference in MPOD increase was observed between treatment groups, these findings suggest that the scleral route may be effective for delivering lutein to the posterior segment and that low-intensity electric currents may enhance its accumulation within chorioretinal tissues [12,27,28]. Preclinical and clinical studies have demonstrated that ocular iontophoresis can increase intraocular drug concentrations in the posterior segment while minimizing systemic exposure, with a favorable safety profile and good tolerability [12,27,28]. Recent translational data further support the feasibility of targeted lutein delivery to the macula via scleral iontophoresis in intermediate AMD [13].

When interpreting these findings in the context of large supplementation trials, it is important to distinguish between systemic approaches aimed at modifying overall nutritional status and localized ocular delivery strategies. In the AREDS2 study, the addition of lutein and zeaxanthin to the original antioxidant formulation was associated with a modest but clinically meaningful reduction in the risk of progression to advanced AMD, particularly in patients with low baseline intake or in those not receiving β-carotene [4,24]. However, AREDS2 and similar trials did not directly compare different delivery routes, nor did they evaluate MPOD as a primary endpoint. Smaller interventional studies focusing specifically on macular pigment have consistently shown that oral supplementation increases MPOD and may improve functional parameters such as contrast sensitivity in early and intermediate AMD [19,20,21,23,29]. Compared with these studies, the present results suggest that both oral and transscleral approaches increase MPOD over a similar follow-up period [12].

In addition to efficacy, practical aspects of treatment selection should be considered. Patients with AMD frequently present with systemic comorbidities, such as cardiovascular disease, hypertension, type 2 diabetes, and dyslipidemia, and are often exposed to polypharmacy [1,2,4]. It is plausible that increasing the number of daily oral interventions, even in the form of supplements, may negatively affect adherence and tolerability. Furthermore, some concomitant medications may interfere with carotenoid absorption, metabolism, or transport [22,30]. In selected patients with potentially reduced systemic carotenoid bioavailability or a high burden of concomitant therapies, approaches that bypass the gastrointestinal tract and systemic circulation may therefore offer practical advantages [12,13]. In this context, transscleral lutein iontophoresis, as a localized and targeted delivery approach, may represent a valuable alternative or adjunct to oral supplementation. A treatment regimen based on intermittent in-office iontophoresis sessions may also improve real-world adherence by shifting the responsibility for administration from the patient to the clinical setting, similarly to other procedure-based ophthalmic therapies [29], although these considerations remain speculative and should be formally evaluated in future comparative studies [29].

Future research should include randomized controlled trials specifically designed to compare oral supplementation, transscleral iontophoresis, and their combination. A three-arm study in patients with intermediate AMD, stratified by baseline MPOD and systemic carotenoid status, with a follow-up of at least 12–24 months, would allow for the evaluation of both short-term changes in MPOD and visual function, as well as long-term structural outcomes [19,29]. Primary endpoints could include the magnitude and stability of MPOD increase and sensitive functional measures such as contrast sensitivity, mesopic and scotopic vision, and dark adaptation. Secondary endpoints may include OCT biomarkers of disease progression and rates of conversion to neovascular AMD [4].

Overall, the findings of this pilot study confirm the effectiveness of oral supplementation in increasing macular pigment levels and suggest that transscleral lutein iontophoresis resulted in comparable increases in MPOD, supporting its potential as an alternative delivery strategy [12,13]. Given the growing body of epidemiological and clinical evidence linking adequate lutein and zeaxanthin intake to reduced AMD risk and improved retinal function [1,2,4,19,20,21,22,30], the development of delivery strategies capable of maximizing retinal carotenoid availability is of considerable clinical interest. In selected patients, particularly those with impaired systemic absorption or high treatment burden, targeted approaches such as iontophoresis may complement conventional supplementation to optimize macular antioxidant protection.

These results should be interpreted in light of several limitations, including the non-randomized design, the relatively small sample size, and the limited 6-month follow-up. While this duration is sufficient to detect short-term changes in MPOD, it does not allow conclusions regarding disease progression or long-term clinical benefit.

In addition, the absence of randomization and adjustment strategies (such as matching or multivariable analysis) limits the interpretability of between-group comparisons. Therefore, any comparison between treatment arms should be considered exploratory and interpreted with caution.

Another important limitation is the lack of functional outcome measures. Parameters such as contrast sensitivity, glare disability, dark adaptation, and mesopic visual performance are particularly relevant in intermediate AMD and may be more sensitive than BCVA in detecting subtle functional changes. The reliance on structural parameters and surrogate biomarkers, such as MPOD, further limits the clinical interpretability of the findings.

Although the between-group difference in MPOD change did not reach statistical significance, a moderate effect size was observed, suggesting a potentially clinically meaningful difference in favor of iontophoresis. This finding should be interpreted with caution, as the absence of an a priori sample size calculation and the relatively small cohort may have limited the statistical power of the study. Therefore, a type II error cannot be excluded.

Larger, adequately powered randomized studies with longer follow-up are needed to confirm these findings, better define optimal treatment protocols, and evaluate the impact of this approach on functional outcomes, including contrast sensitivity, dark adaptation, and patient-reported visual function, as well as its potential role in modifying disease progression from intermediate to advanced AMD [1,19,20,21,31].

## 5. Conclusions

In this prospective, non-randomized pilot study, both oral macular carotenoid supplementation and transscleral lutein iontophoresis were associated with a significant increase in macular pigment optical density (MPOD) in eyes with intermediate age-related macular degeneration, while best-corrected visual acuity and central macular thickness remained stable over the 6-month follow-up. No treatment-related safety concerns were observed.

No statistically significant differences in MPOD change were observed between treatment groups, suggesting that, within the limitations of this small, non-randomized pilot study, transscleral iontophoresis may represent a feasible alternative strategy for carotenoid delivery, with short-term effects on MPOD comparable to those of oral supplementation. These findings are exploratory and should be interpreted with caution, as the study was not powered to detect modest between-group differences and did not include detailed functional endpoints. From a clinical perspective, transscleral lutein iontophoresis could be considered as a potential adjunct or alternative to oral supplementation in selected patients, particularly those with systemic comorbidities or a high treatment burden, where adherence or absorption may be suboptimal. The main limitations of this study include the non-randomized design, the relatively small cohort, the short duration of follow-up, and the absence of a priori sample size calculation, which may have limited the ability to detect differences between treatments and to assess long-term outcomes. In addition, although MPOD is a well-established biomarker of macular carotenoid status, its relationship with functional outcomes and disease progression requires further clarification.

Larger, randomized studies with longer follow-up are warranted to confirm these preliminary findings, to better define the role of transscleral iontophoresis, and to determine whether the observed changes in MPOD translate into meaningful clinical benefits, including functional outcomes and reductions in advanced AMD.

## Figures and Tables

**Table 1:** Sixty patients (60 eyes) completed the 6-month follow-up. Baseline demographic and clinical parameters were comparable between the two cohorts (all *p* > 0.05).

Parameters	Supplementation (*n* = 30)	Iontophoresis (*n* = 30)	*p*-Value
Age	71.4 ± 6.8	72.1 ± 7.2	0.68
BCVA (logMAR)	0.18 ± 0.09	0.17 ± 0.08	0.74
MPOD	0.41 ± 0.10	0.40 ± 0.11	0.81
CMT (µm)	276 ± 21	279 ± 24	0.59

## Data Availability

The data presented in this study are available on reasonable request from the corresponding author.

## Abbreviations

The following abbreviations are used in this manuscript:

AMD	Age-Related Macular Degeneration
iAMD	Intermediate Age-Related Macular Degeneration
MPOD	Macular Pigment Optical Density
BCVA	Best-Corrected Visual Acuity
CMT	Central Macular Thickness
OCT	Optical Coherence Tomography
EDI-OCT	Enhanced Depth Imaging Optical Coherence Tomography
ETDRS	Early Treatment Diabetic Retinopathy Study
logMAR	Logarithm of the Minimum Angle of Resolution
SD	Standard Deviation
RPE	Retinal Pigment Epithelium
AREDS2	Age-Related Eye Disease Study 2
du	Density Units
